# Secreted Wnt antagonists in scrub typhus

**DOI:** 10.1371/journal.pntd.0009185

**Published:** 2021-04-29

**Authors:** Thor Ueland, Elisabeth Astrup, Kari Otterdal, Tove Lekva, Jeshina Janardhanan, John A. J. Prakash, Kurien Thomas, Annika E. Michelsen, Pål Aukrust, George M. Varghese, Jan K. Damås

**Affiliations:** 1 Research Institute of Internal Medicine, Oslo University Hospital Rikshospitalet, Oslo, Norway; 2 Faculty of Medicine, University of Oslo, Oslo, Norway; 3 K.G. Jebsen Thrombosis Research and Expertise Center, University of Tromsø, Tromsø, Norway; 4 Institute of Clinical Medicine, Akershus University Hospital, Lørenskog, Norway; 5 Department of Medicine and Infectious Diseases, Christian Medical College, Vellore, Tamil Nadu, India; 6 Department of Microbiology, Christian Medical College, Vellore, Tamil Nadu, India; 7 Department of Medicine, Christian Medical College, Vellore, Tamil Nadu, India; 8 Section of Clinical Immunology and Infectious Diseases, Oslo University Hospital Rikshospitalet, Oslo, Norway; 9 Department of Clinical and Molecular Medicine, Norwegian University of Science and Technology, Trondheim, Norway; 10 Department of Infectious Diseases, St. Olavs Hospital, Trondheim, Norway; Mahidol University, THAILAND

## Abstract

**Background:**

The mechanisms that control local and systemic inflammation in scrub typhus have only been partially elucidated. The wingless (Wnt) signaling pathways are emerging as important regulators of inflammation and infection, but have not been investigated in scrub typhus.

**Methodology/Principal findings:**

Plasma levels of secreted Wnt antagonists (i.e. DKK-1, sFRP-3, WIF-1 and SOST) were analyzed in patients with scrub typhus (n = 129), patients with similar febrile illness without *O*. *tsutsugamushi* infection (n = 31), febrile infectious disease controls, and in healthy controls (n = 31) from the same area of South India, and were correlated to markers of inflammation, immune and endothelial cell activation as well as for their association with organ specific dysfunction and mortality in these patients. We found i) Levels of SOST and in particular sFRP-3 and WIF-1 were markedly increased and DKK-1 decreased in scrub typhus patients at admission to the hospital compared to healthy controls. ii) In recovering scrub typhus patients, SOST, sFRP-3 and WIF-1 decreased and DKK-1 increased. iii) SOST was positively correlated with markers of monocyte/macrophage and endothelial/vascular activation as well as with renal dysfunction and poor outcome iv) Finally, regulation of Wnt pathways by *O*. *tsutsugamushi* in vitro in monocytes and ex vivo in mononuclear cells isolated from patients with scrub typhus, as evaluated by gene expression studies available in public repositories, revealed markedly attenuated canonical Wnt signaling.

**Conclusions/Significance:**

Our findings suggest that scrub typhus is characterized by attenuated Wnt signaling possibly involving dysregulated levels of several secreted pathway antagonists. The secreted Wnt antagonist SOST was strongly associated with renal dysfunction and poor prognosis in these patients.

## Author summary

Scrub typhus, a systemic infection caused by *Orientia tsutsugamushi* is manifested by fever and multiple organ involvement with significant mortality if untreated. *O*. *tsutsugamushi* infects endothelial cells triggering inflammatory responses in endothelial and monocyte-derived macrophages. The wingless (Wnt) pathways are important regulators of the interaction between microbes and the immune system promoting inflammatory and anti-inflammatory responses. Wnt signaling is regulated by multiple extracellular secreted proteins that are readily measurable and may reflect activity in the Wnt pathways. We measured plasma levels of the secreted Wnt modulators in patients with scrub typhus and infectious controls and correlated them with markers of inflammation and immune activation. We also evaluated the regulation of Wnt pathways by *O*. *tsutsugamushi in vitro* in monocytes and *ex vivo* in mononuclear cells isolated from patients in microarray experiments available in public repositories. Our study suggests a pathogenic role for dysregulated Wnt signaling during acute *O*. *tsutsugamushi* infection.

## Introduction

Scrub typhus, a systemic infection caused by the vector-borne obligate intracellular gram-negative vector-born bacteria *Orientia tsutsugamushi* (*O*. *tsutsugamushi*), is endemic in the Asia-Pacific resulting in estimated one million new infections each year. It is manifested by fever and multiple organ involvement with significant mortality if untreated [1]. *O*. *tsutsugamushi* infects endothelial cells triggering a wide range of inflammatory responses in endothelial and non-endothelial cells such as monocyte-derived macrophages, representing both beneficial (i.e. anti-microbial) and detrimental (e.g. tissue destruction) effects in the infected host [2,3]. Exposure of monocytes and macrophages to *O*. *tsutsugamushi in vitro* regulates multiple inflammatory pathways with a similar pattern *ex vivo* in mononuclear cells isolate from patients [4,5]. It has been shown that scrub thyphus is characterized by dysregulated circulating levels of a wide range of inflammatory markers reflecting activation of monocyte/macrophage and endothelial cells [6,7].

The wingless (Wnt) pathways are emerging as important regulators of the interaction between microbes and the immune system promoting inflammatory, but also anti-inflammatory responses [8–12]. Indeed, pathogen manipulation of Wnt signaling has been linked to immune evasion involving anti-inflammatory mechanisms [8,13]. Thus, the secreted Wnt antagonist Dickkopf-1 (DKK-1), which promotes inflammation by inhibiting canonical Wnt activation [14], is down-regulated by *Rickettsia conorii* (*R*. *conorii)*, potentially contributing to dampen the immune response against the invading bacteria [15]. Manipulation of the canonical Wnt pathway may also contribute to immune evasion by limiting apoptosis and phagocytosis [8,10,11,13]. In addition, activation of non-canonical signaling may play a role during infection [10]. In particular, Wnt5a has been identified as a potent inducer of inflammatory responses in monocytes and macrophages that restrict infection by different mechanisms [10].

In addition to DKKs, activation of Wnt signaling is regulated by multiple other extracellular secreted proteins including members of the secreted frizzled-related protein (SFRP), Sclerostin (SOST) and Wnt inhibitory factor families (WIF) [14,16]. Furthermore, while Wnt ligands circulate at low and undetectable levels, these antagonists are readily measurable and may represent a negative feedback loop to limit Wnt pathway activity, thereby reflecting activity in the Wnt pathways [14,16].

To the best of our knowledge circulating Wnt-related proteins have not been investigated in Scrub typhus. We hypothesized that plasma levels of the secreted Wnt modulators sFRP-3, DKK-1, SOST and WIF-1 would be regulated in scrub typhus and correlate with markers of inflammation and immune activation. We also evaluated the regulation of Wnt pathways by *O*. *tsutsugamushi in vitro* in monocytes and *ex vivo* in mononuclear cells isolated from patients in microarray experiments available in public repositories [4,5].

## Materials and methods

### Ethics statement

Blood samples from patients and controls were collected after obtaining informed and written consent from each participant. If the patient was not in a state to give consent such as an unconscious state or on a ventilator and in the case of patients <18 years of age, written consent was obtained from next of kin or parent as appropriate. The study was approved by the local ethic committees; in India by the CMC, IRB & EC (Institutional Review Board $ Ethic Committee, Christian Medical College, Vellore) and the Health Ministry Screening Committee, ICMR (Indian Council of Medical Research), in Norway by the Regional Committee for Medical and Health Research Ethics. It was conducted according to the ethical guidelines from the Helsinki declaration.

### Patients and controls

Patients >15 years of age admitted to Christian Medical College, Vellore, Tamil Nadu, India between November 2009 and February 2011 with suspected scrub typhus were considered for inclusion in to the study. All the patients with confirmed diagnosis of scrub typhus based on a positive IgM enzyme immune-assay (EIA) test were included as cases. The scrub typhus patients were further divided into subgroups according to disease severity. Those with no organ dysfunction were considered to have mild disease, those with one organ dysfunction moderate, while two or more organ dysfunctions were defined as severe disease. Organ dysfunction was defined as follows: Renal dysfunction, creatinine ≥2.5 mg/dl; hepatic dysfunction, bilirubin (total) ≥2.5 mg/dl, pulmonary dysfunction: bilateral pulmonary shadows on chest X-rays with moderate or severe hypoxia (PaO2/FiO2 <300 mmHg/PaO2 <60 mmHg/SpO2 <90%), cardiovascular dysfunction: systolic blood pressure <80 mmHg despite fluid resuscitation and central nervous system dysfunction: significant altered sensorium with Glasgow Coma Scale (GCS) ≤8/15. The patients confirmed to have scrub typhus was treated with doxycycline with or without azithromycin. Treatment including mechanical ventilation and vasoactive agents was decided by the treating physician as per protocol.

Two control groups were included. One group was patients admitted with acute febrile illness, but confirmed to have an alternate infection with negative scrub typhus EIA. Of these patients 6 were dengue fever, 4 typhoid fever, 3 influenza, 2 tuberculosis, 2 acute encephalitis, 1 aseptic meningitis, 1 leptospirosis, 1 pneumonia, 1 liver abscess, 1 urosepsis, 1 rubella, 1 viral hepatitis, and 7 had infectious disorders of uncertain etiology. In addition, 31 healthy controls (14 female, 17 male) recruited from the same area of South India as the patients were also included in the study.

### Blood sampling protocol

Blood samples were collected at first presentation, before specific treatment, and at follow-up (1–2 weeks after the initial sample). Peripheral venous blood was drawn into pyrogen-free, vacuum blood collection tubes with EDTA as anticoagulant, centrifuged within 30 minutes at 2000 g for 20 minutes to obtain platelet-poor plasma, and the obtained samples were stored in multiple aliquots at −80°C until analysis. All samples were thawed less than three times.

### Microbiological diagnosis

Scrub typhus IgM EIA was performed on serum samples using the Scrub Typhus Detect (InBios International, Inc., Seattle, WA). The IgM EIA test was initially standardized using serum samples from healthy blood donors and the OD cutoff of 0.5 was taken 3 SD from the mean. Further validation was done using known scrub typhus sera (confirmed by PCR/immunofluorescence) and sera from patients with other diseases like malaria and enteric fever and healthy controls. We also used a positive and a negative control provided in the kit as well as an in-house positive control for every run. This test has a sensitivity and specificity of >90% [17]. A subset of patients also had further confirmation by PCR on eschar samples as described [18,19].

### Enzyme immunoassays

Plasma levels of sFRP3, DKK1, WIF1 and SOST (validation [20]) were measured by EIAs obtained from R&D Systems (Minneapolis, MN). The intra- and inter-assay coefficients of variations were <10% for all EIAs. To further minimize run-to-run variability, serial samples from a given individual were analyzed on the same tray. Plasma levels of soluble (s) vascular adhesion molecule-1 (sVCAM)-1, YKL-40, sCD14, sCD163 and macrophage inhibitory factor-1 (MIF-1) have been reported previously [6].

### Evaluation of Wnt family expression from public databases

SOST mRNA expression were obtained from the protein atlas (proteinatlas.org/). mRNA expression from relevant studies of *O*. *tsutsugamushi stimulation in vitro* in monocytes and *ex vivo* in mononuclear cells isolated from patients in microarray experiments available in public repositories [4,5].

### Statistics

Differences in inflammatory markers in patients with scrub typhus, acute infection controls and healthy controls were compared with the Kruskal-Wallis test a priori and if significant, the Mann Whitney U test was used to compare the different groups. Paired differences (i.e., within scrub typhus group) were compared using the Wilcoxon signed-rank test. Bivariate correlations between secreted Wnt antagonists and other clinical and biochemical variables was assessed by Spearman correlation.

Predictors of disease severity were identified by stepwise linear regression (0.10 to enter, 0.15 to exclude) including the inflammatory markers and creatinine, albumin, bilirubin, alkaline-phosphatase, age and gender. Variables were log transformed prior to regression. Associations between inflammatory markers and mortality (n  =  7) were investigated by receiver operation curve (ROC) analysis. P values are two-sided and considered significant when <0.05.

## Results

### Plasma levels of secreted Wnt antagonists at baseline and during follow-up in patients with scrub typhus (Fig 1)

**Fig 1:**
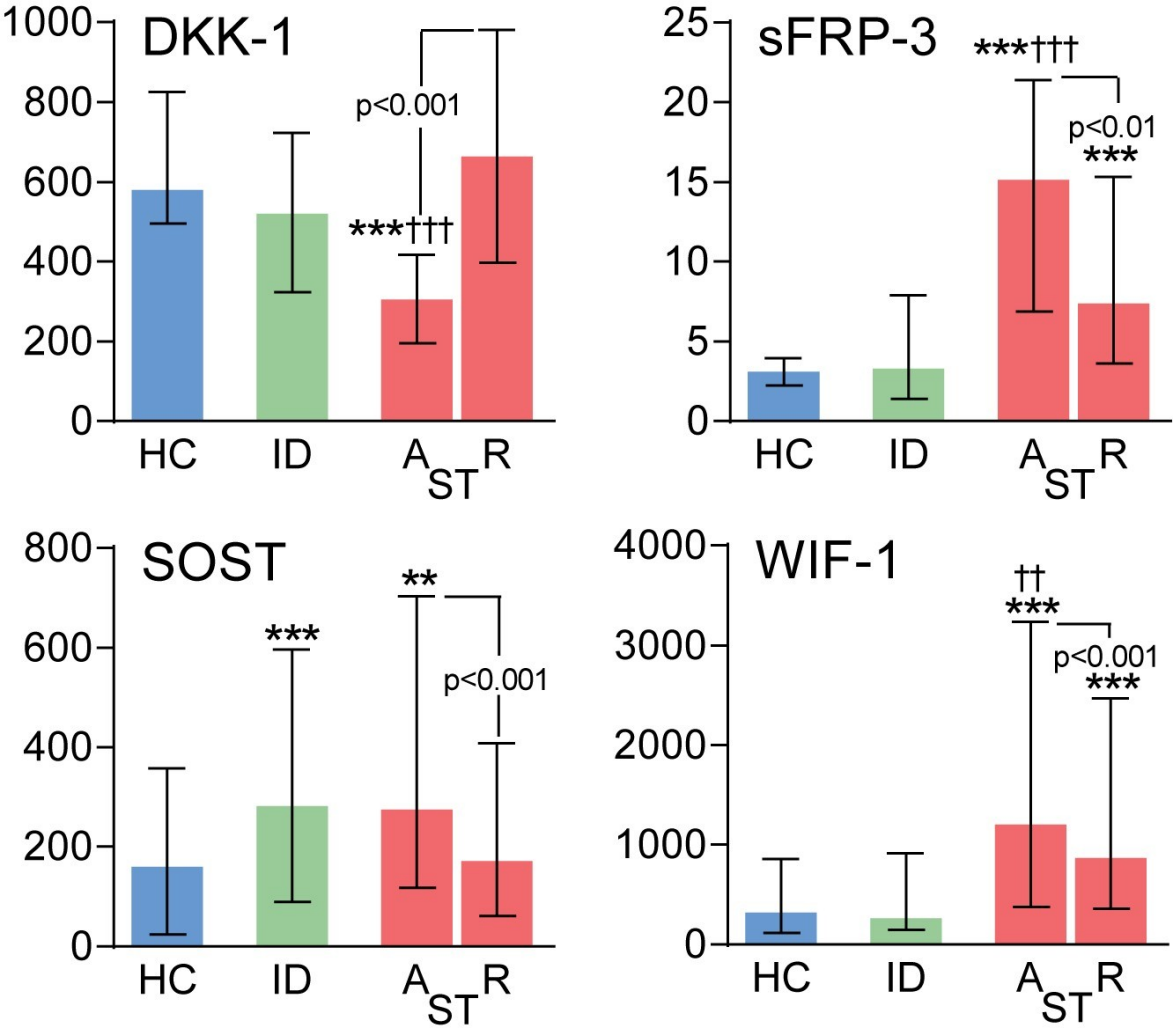
Plasma levels of secreted Wnt antagonists in scrub typhus patients and healthy controls. Levels of Dickopff-1 (DKK-1), secreted frizzled related protein-3 (sFRP-3), sclerostin (SOST) and Wnt inhibitory factor (WIF-1) in scrub typhus patients (n = 129) on admission (A) and at recovery (R) as well as comparative levels in healthy controls (HC, n = 31) and infectious disease controls (ID, n = 31). Data are given as medians and 25th, 75th percentiles. *p<0.05, **p<0.01 and ***p<0.001 versus healthy controls; ^††^p < 0.01 and ^†††^p < 0.001 versus infectious disease controls. Comparisons between levels at admission and recovery are also shown with p-values.

Plasma levels of secreted Wnt antagonists were analyzed in patients with scrub typhus (n = 129), patients with similar febrile illness without *O*. *tsutsugamushi* infection (n = 31, febrile infectious disease controls, see methods), and in healthy controls (n = 31) from the same area of South India (Table 1). Levels of SOST and in particular sFRP-3 and WIF-1 were markedly increased in scrub typhus patients at admission to the hospital compared to healthy controls and notably for sFRP-3 and WIF-1, also when compared to admission levels in infectious controls (Fig 1). Notably, an opposite pattern was seen for DKK-1 that was decreased in patients with scrub typhus and in infectious controls compared to healthy individuals. At recovery (median 17 days, range 5–135) after the initial sample, scrub typhus patients had a significant decrease in SOST and increase in DKK-1 levels compared to admission levels, reaching levels comparable to healthy controls. A significant decrease in sFRP-3 and WIF-1 was also observed at recovery, but levels remained elevated compared to healthy controls.

**Table 1:** Characteristics of patients with scrub typhus according to disease severity and infectious disease controls.

	Infectious controls (n = 31)	Mild disease (n = 51)	Moderate disease (n = 37)	Severe disease (n = 41)
Age (mean±SD)	35±18	50±14	45±17	40±16
Gender (male/female)	17/14	24/27	25/12	17/24
Renal dysfunction, n (%)	2 (7)	0	6 (16)	15 (37)
Hepatic dysfunction, n (%)	4 (13)	0	7 (19)	24 (59)
CNS dysfunction, n (%)	1 (3)	0	0	1 (2.4)
Respiratory dysfunction, n (%)	1 (3)	0	10 (27)	26 (63)
Circulatory dysfunction, n (%)	4 (13)	0	5 (14)	20 (49)
Creatinine, mg/dL	1.2 (0.9, 1.4)	1.2 (1.0, 1.7)	1.2 (1.0, 1.7)	1.8 (1.1, 3.3)
eGFR	74 (59, 85)	60 (46, 81)	65 (38, 80)	36 (19, 72)
Total bilirubin, mg/dL	0.8 (0.4, 1.6)	0.7 (0.5, 1.0)	1.4 (0.9, 2.1)	2.8 (1.3, 6.0)
Total s-protein,	6.6 (6.3, 7.1)	6.8 (6.3, 7.4)	6.4 (6.0, 7.1)	6.1 (5.8, 6.7)
Albumin, g/dL	3.4 (3.0, 3.8)	3. 0 (2.6, 3.3)	2.7(2.4, 3.0)	2.6 (2.4, 2.7)
AST, U/L	55 (46, 145)	123 (84, 175)	143 (96, 201)	190 (112, 247)
ALT, U/L	36 (20, 93)	65 (45, 121)	84 (48, 109)	65 (52, 117)
Alkaline phosphatase, U/L	112 (74, 182)	122 (85, 176)	158 (120, 230)	219 (170, 306)

Data for the biochemical parameters in serum are given as medians (25^th^, 75 percentiles). AST, aspartate aminotransferase; ALT, alanine aminotransferase.

### Plasma levels of secreted Wnt antagonists in relation to markers of inflammation and endothelial cell activation

As shown in Table 2, SOST was robustly positively correlated with general markers of inflammation as reflected by CRP, markers of monocyte/macrophage activation (sCD163, sCD14 and MIF) and markers of endothelial activation (sVCAM-1). Except for a strong correlation between SOST and sCD14 in infectious controls, these correlations were not present in the control groups. DKK-1 and sFRP-3 displayed similar, but opposite, correlations with markers of monocyte/macrophage and endothelial cell activation in infectious controls and scrub typhus with negative associations for DKK1, and positive associations for sFRP-3. No correlation with these markers was observed for WIF-1.

**Table 2:** Plasma levels of secreted Wnt antagonists in relation to markers of inflammation, immune and endothelial cell activation in healthy controls (HC), infectious controls (IC) and patients with scrub typhus (ST).

		DKK-1			sFRP-3			SOST			WIF-1	
Grp:	HC	IC	ST	HC	IC	ST	HC	IC	ST	HC	IC	ST
CRP	-0.53**	0.10	-0.08	-0.09	-0.29	-0.07	0.20	0.19	0.50**	0.07	-0.27	-0.15
CD163	0.11	-0.51**	-0.31**	0.36*	0.28	0.17	0.31	0.15	0.32**	0.21	0.09	0.15
sCD14	0.10	-0.37*	-0.13	0.08	0.30*	0.19*	0.20	0.50**	0.36**	0.25	0.23	0.08
MIF	0.07	0.12	0.11	0.16	-0.19	-0.07	0.26	-0.26	0.39**	0.30	-0.26	-0.15
YKL40	-0.23	-0.11	-0.05	0.22	0.16	-0.03	0.20	0.30	0.49**	-0.07	-0.06	-0.10
VCAM1	0.06	-0.57**	-0.21*	0.11	0.57**	0.25**	0.24	0.33	0.32**	-0.02	0.35	0.13

Data are given as rho and * represents p-value

*p<0.05

**p<0.001. DKK, Dickkopf; sFRP

secreted frizzled related protein; SOST, sclerostin; WIF, Wnt inhibitory factor

### Plasma levels of secreted Wnt antagonists in relation to severity and mortality

During a median follow-up of 27 days (range 6–137 days), 7 patients died. As shown in Table 3, excluding SOST, the Wnt antagonists were not associated with severity, organ specific dysfunction or outcome. In contrast, high SOST levels were associated with renal dysfunction with approximately 3 times higher SOST levels in patients with renal dysfunction (median 608 [25^th^ percentile: 378, 75^th^ percentile: 775]) compared to those without (234 [135, 388] p<0.001) and a similar increase in those who died (681 [431, 1287]) compared to survivors (242 [154, 402] p<0.001).

**Table 3:** Plasma levels of secreted Wnt antagonists in relation to severity and outcome.

	DKK-1	sFRP-3	SOST	WIF-1
Severity, H (p)	0.9 (0.40)	0.9 (0.88)	1.2 (0.48)	1.0 (0.87)
Renal dysfunction, H (p)	1.1 (0.62)	1.0 (0.55)	**2.6 (<0.001)**	1.0 (0.34)
Hepatic dysfunction, H (p)	0.9 (0.49)	1.0 (0.95)	1.0 (0.76)	0.9 (0.70)
Respiratory dysfunction, H (p) (p)	0.8 (0.11)	1.0 (0.64)	1.0 (0.92)	-0.7 (0.46)
Circulatory dysfunction, H (p)	0.7 (0.015)	1.3 (0.13)	1.3 (0.22)	1.4 (0.080)
Death, H (p)	0.9 (0.67)	1.2 (0.66)	**2.8 (<0.001)**	1.1 (0.69)

H statistic from Kruskal Wallis test

DKK, dickkopf; sFRP secreted frizzled related protein; SOST, sclerostin; WIF, Wnt inhibitory factor

### Associations between plasma levels of SOST, inflammation, renal function and adverse outcome

**Fig 2:**
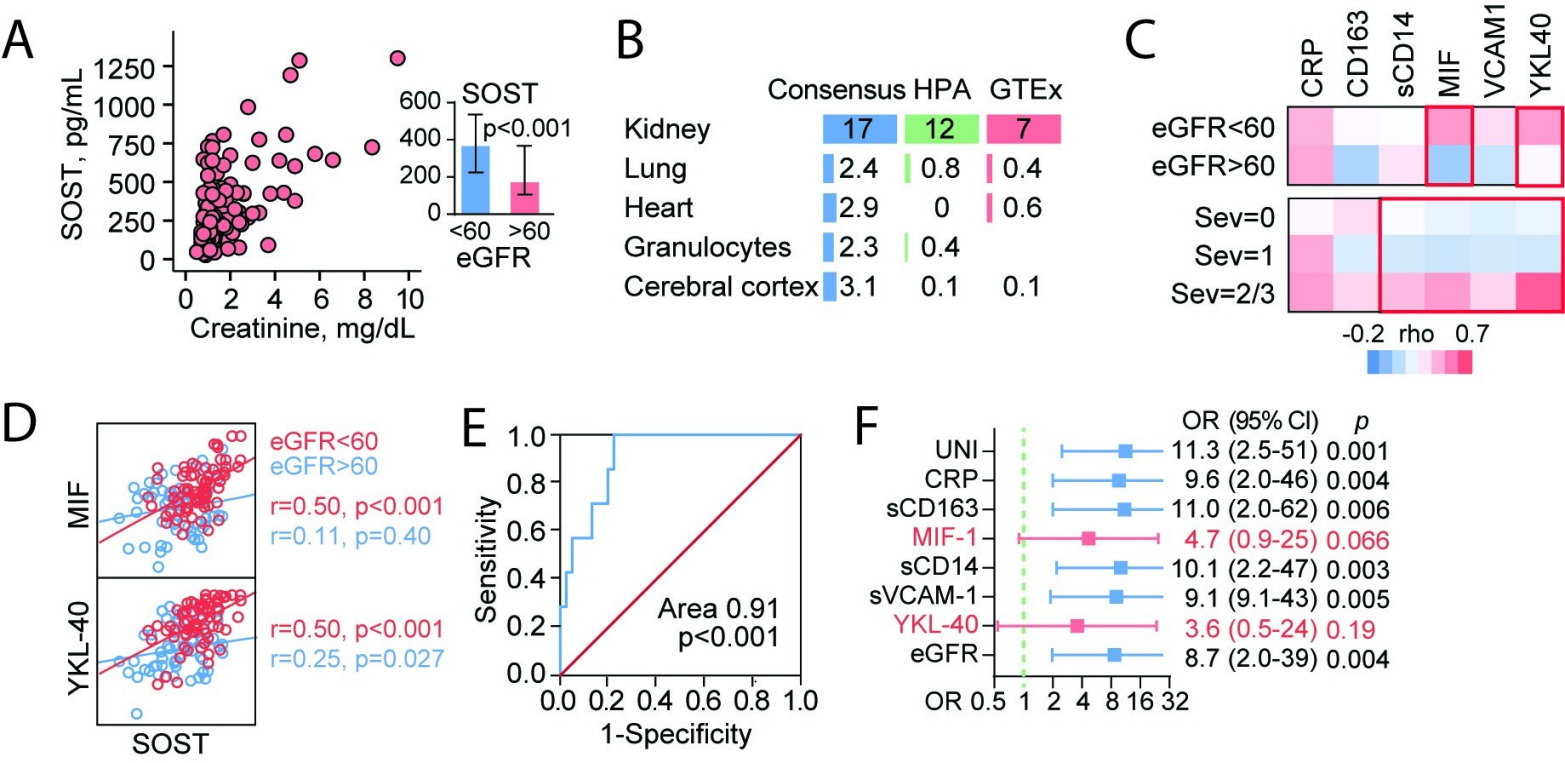
Plasma sclerostin (SOST) and associations with renal function, inflammation, and adverse outcome. (A) Correlation between SOST and creatinine in patients with scrub typhus. The small graph shows SOST levels according to renal dysfunction (i.e. eGFR<60). (B) Tissue expression data from human protein atlas (proteinatlas.org/). (C) Association between SOST and inflammatory markers according to kidney function (eGFR<60, n = 53; eGFR>60, n = 76) and severity (Mild Sev = 0, n = 51; Moderate, Sev = 1, n = 37; Severe, Sev = 2/3, n = 41). (D) Correlation (Spearman) between plasma SOST and MIF and YKL-40 according to kidney function (eGFR<60). (E) Receiver operating characteristic analysis showing associations between mortality and SOST levels in scrub typhus patient on admission. (F) Logistic regression showing odds ratio (OR) for SOST in relation to all-cause death (UNI). The impact of each inflammatory marker and eGFR on this association is shown. SOST is presented as log/SD.

The strong association between SOST and renal dysfunction and adverse outcome prompted us to investigate this association in more detail. First, we observed a strong positive correlation between SOST levels in patients with scrub typhus and creatinine and accordingly, patients with eGFR<60 had markedly higher SOST levels (Fig 2A). Evaluation of tissue mRNA expression of SOST from public databases (human protein atlas, Consensus, HPA and GTEx datasets) revealed an abundance of SOST in renal tissues compared to other tissues (Fig 2B). As shown in Table 2, in scrub typhus patients SOST correlated well with markers of inflammation and endothelial cell activation and further evaluation of these correlations revealed that these associations were driven by, or augmented, in patients with more severe disease or with kidney damage (Fig 2C). The correlation between SOST and YKL-40 and MIF is presented in Fig 2D and shows a markedly stronger association in patients with poor kidney function (i.e. eGFR<60). The elevated SOST in patients who died, as reported in Table 2, gave a very good discrimination with an AUC of 0.91 (Fig 2E) and a OR of 11.3 in univariate analysis, although the CI’s were very large (Fig 2F). Fig 2F shows the impact of the different inflammatory markers on SOSTs association with outcome. While CRP, sCD163, sCD14 and sVCAM-1, had minimum impact on this association, the addition of MIF-1 and YKL-40 markedly attenuated the association between SOST and death.

### Regulation of Wnt pathways by *O*. *tsutsugamushi*, data from public repositories

**Fig 3:**
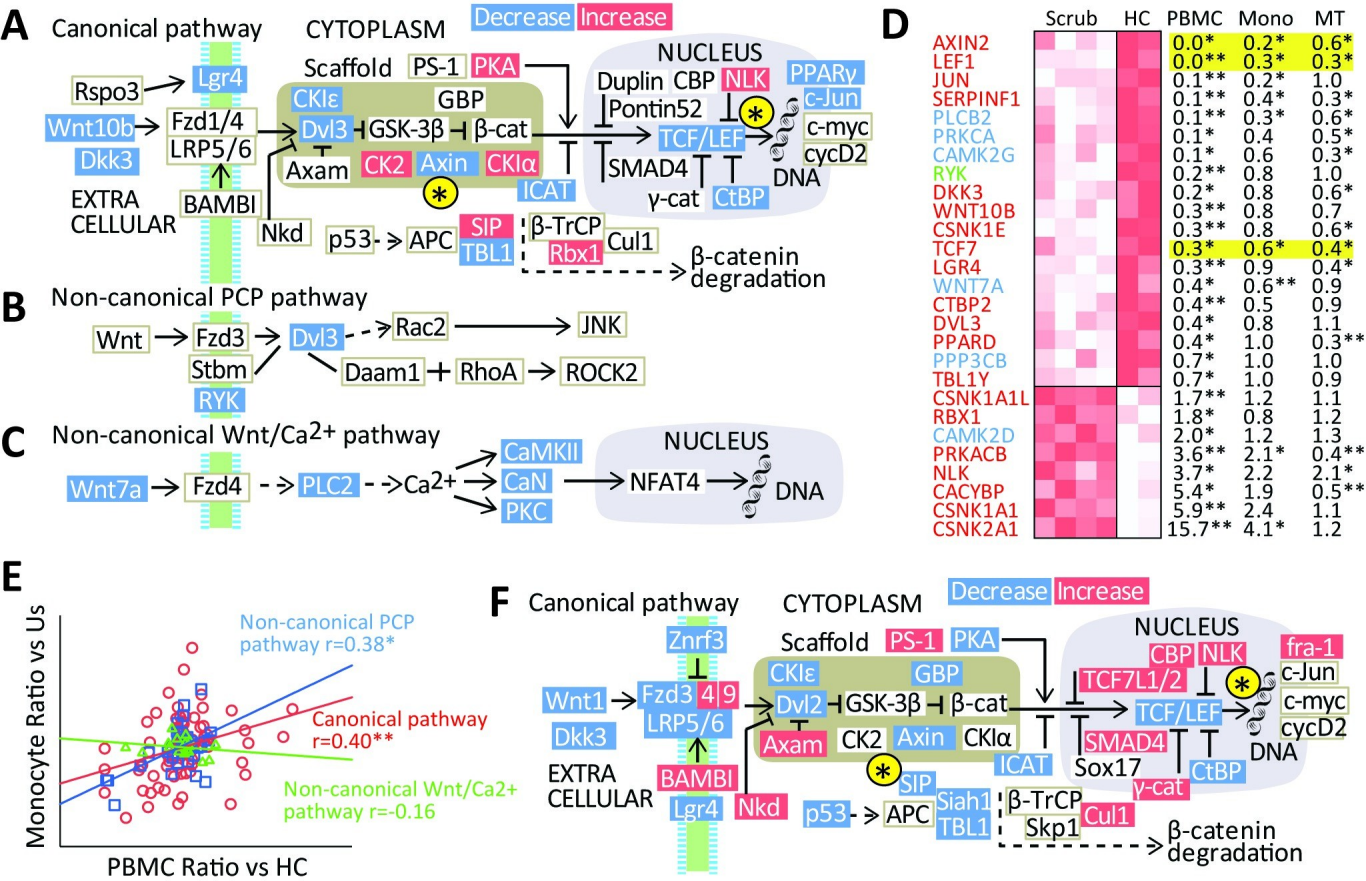
Regulation of Wnt pathways by *O*. *tsutsugamushi* in mononuclear cells and PBMC, data from public repositories. Differences in Wnt pathway gene expression in PBMC between patients with scrub typhus and healthy controls (HC). Significant results (FDR adjusted p-values) are indicated in red/blue (for increased/decreased mRNA expression). The figure summarizes relevant genes for the (A) canonical,(B) non-canonical PCP pathway and (C) non-canonical Wnt/Ca2+ pathway. The figure is based on the Wnt signaling pathway in the KEGG database. (D) Heatmap over the regulated mRNA in the canonical pathway (red text), non-canonical PCP pathway (green) and non-canonical Wnt/Ca^2+^ pathway (blue) in PBMC between patients with scrub typhus (n = 4) and healthy controls (n = 2). Red indicates a higher expression. To the right is a table over regulated mRNA between patients with scrub typhus and healthy controls (PBMC), in monocytes (Mono) infected with *O*. *tsutsugamushi* compared to non-infected cells (n = 4 in each group) and in patients with scrub typhus compared to murine typhus (MT, Scrub n = 4, typhoid n = 7). *p<0.05, **p<0.01. (E) Correlation between fold change in PBMC (scrub patients vs. controls) and *O*. *tsutsugamushi* infected mononuclear cells. (F) Regulation of the canonical Wnt pathways by *O*. *tsutsugamushi* in mononuclear cells.

We next probed regulation of Wnt pathways by *O*. *tsutsugamushi in vitro* in monocytes and *ex vivo* in mononuclear cells isolated from patients in microarray experiments available in public repositories [4,5]. Fig 3A–3C shows regulation of the Wnt pathways as outlined by KEGG (Kyoto encyclopedia of genes and genomes) in PBMC from patients with scrub typhus. The overall impression is an attenuated Wnt activation in these patients, in particular for the canonical pathway. Thus, prominent downstream readout of canonical Wnt signaling such as AXIN2, TCF7, LEF1 as well as several transcriptional targets such as PPARγ and c-Jun were markedly decrease compared to healthy controls. These data are also shown as a heatmap in Fig 3D. The fold change in regulated Wnt mRNAs in PBMC from patients with scrub typhus is shown to the right of the heatmap, and corresponding regulation in monocytes infected with *O*. *tsutsugamushi* compared to non-infected cells (Fig 3F), as well as in patients with scrub typhus compared to typhoid typhus are shown. The down regulation of downstream readouts of canonical Wnt signaling such as AXIN2, TCF7 and LEF1 in scrub typhus patients was quite similar compared to controls, in monocytes infected with *O*. *tsutsugamushi* and compared to patients with typhoid typhus. The correlations between Wnts regulated in PBMC from scrub patients and monocytes infected with *O*. *tsutsugamushi* was good for members of the canonical and non-canonical PCP pathway, but not for the non-canonical Wnt/Ca2+ pathway (Fig 3E).

## Discussion

Our study shows that scrub typhus is characterized by a marked dysregulation of secreted Wnt antagonists during the acute phase, differing not only from healthy controls in the same region of South India, but also from infectious disease controls admitted to the hospital with a febrile illness and similar symptoms as the scrub typhus patients. In particular, sFRP3 and WIF-1 were elevated and DKK1 was decreased in scrub patients and normalized during follow-up. A more modest elevation was observed for SOST, but markedly higher levels were found in scrub patients with renal dysfunction or who died, and correlated strongly with markers reflecting monocyte/macrophage activation in these sub-groups. Our findings suggest that scrub typhus is characterized by attenuated Wnt signaling possibly involving dysregulated levels of several secreted pathway antagonists.

We have previously shown that DKK-1, which may promote inflammation by inhibiting canonical Wnt activation [14], is down-regulated by *R*. *conorii*, possibly to attenuate inflammatory responses and avoid innate immune pressure in the host [15]. Similar effects have been reported for *Salmonella*, *Chlamydia*, *Rickettsia*, and *Mycobacteria* suggesting that canonical pathway activation may contribute to an anti-inflammatory state which mediates immunosuppression and prolongs infection [13]. Manipulation of host immune mechanisms involving anti-inflammatory mechanisms has also been reported for *O*. *tsutsugamushi* [21,22]. Herein we show that DKK-1 was negatively correlated with both markers of immune activation (i.e. sCD163 and sCD14) and vascular inflammation (i.e. VCAM1) in patients with scrub typhus and infectious controls suggesting a common mechanism. However, evaluation of *in vitro* studies in monocytes and *ex vivo* studies in mononuclear cells isolated from patients with scrub typhus [4,5] revealed markedly down-regulated readouts of canonical Wnt signaling, such as TCF7, LEF and AXIN2 as well as several transcriptional targets such as PPARγ and c-Jun compared to both healthy controls and patients with murine typhus. Thus, the low DKK-1 and increased sFRP-3 and WIF-1 levels in scrub patients probably do not reflect reduced inhibition of canonical pathways, but rather a suppressive effect of *O*. *tsutsugamushi* on canonical Wnt signaling with reduced transcriptional activity on downstream targets such as DKK-1. We have observed a similar imbalance of Wnt antagonists with attenuated DKK1 and enhanced sFRP-3 in relation to poor virological outcome following treatment for CMV disease [23]. CMV infection may dysregulate canonical Wnt signaling through effects on endogenous β-catenin with reduced levels of downstream genes such as DKK-1 [24]. Taken together, we hypothesize that increased Wnt antagonism and decreased expression of Wnt ligands could contribute to attenuated Wnt signaling during *O*. *tsutsugamushi* infection.

Despite the substantial dysregulation of circulating DKK-1, sFRP-3 and WIF-1 in scrub typhus patients, these antagonists were not associated with poor outcome. In contrast, SOST, which was increased in both scrub patients and infectious controls, was strongly associated with poor prognosis in scrub typhus patients and were strongly correlated with markers of macrophage and endothelial cell activation. SOST has mainly been investigated for its negative impact on bone formation by binding to Wnt co-receptors LRP5/6 and blocking signaling through Frizzled receptors, thereby inhibiting canonical Wnt signaling [25]. Osteocytes are reported as primary source of SOST, however evaluation of expression data suggest abundant expression in the kidneys compared to other tissues. In support of the strong correlation between SOST and creatinine and higher levels in scrub patients with poor kidney function, SOST is closely linked to chronic kidney disease (CKD) [25], although it is unclear if raised levels are due reduced renal clearance or extraskeletal production [26]. SOST levels are also markedly elevated in critically ill patients, linked to renal or hepatic organ failure [27]. In CKD, SOST levels correlate with vascular health and high levels are associated with poor outcome in some [28,29], but not all studies [30]. Although, SOST levels may be driven by PTH metabolism in CKD, circulating levels correlated with markers of endothelial dysfunction and inflammation [29,31], similar to the present study. Moreover, the association between SOST and inflammatory markers in our study was restricted to patients with severe disease, and a markedly stronger association between SOST and MIF and YKL-40 was observed in patients with kidney dysfunction. Furthermore, the association between SOST and outcome was markedly mitigated when these two markers were included in the regression model. We have previously reported that YKL-40 and MIF may contribute to severity and clinical outcome in scrub typhus, possibly related to involvement in endothelial cell activation and adhesion and migration of vascular cells [6]. There is sparse literature on the effects of SOST on vascular cells, however SOST has been demonstrated to stimulate angiogenesis in endothelial cells and act as a chemoattractant for the recruitment of monocytes [32]. Taken together, we hypothesize that SOST could be involved in vascular dysfunction in severely ill scrub typhus patients, involving effects on monocytes/macrophages and endothelial cells.

The present study has some limitations such as few patients with fatal events and lack of data from Wnt members within the tissues at the site of infection. Interpretation of differentially expressed genes from signaling pathways is challenging since most intracellular components are not exclusive, but shared by multiple pathways and possibly more dependent on phosphorylation status than transcription levels. Furthermore, the results from the microarray experiments must be interpreted with caution as only a very small portion of circulating mononuclear cells are infected with O. tsutsugamushi in patients with scrub typhus. They may however aid in identifying dysregulated elements of a pathway that can be evaluated in detail in more relevant cells and tissues in following studies. Also, lack of mechanistic data means that the correlation data should be interpreted with caution. However, our data suggest a pathogenic role for dysregulated Wnt signaling during acute *O*. *tsutsugamushi* infection in humans.
